# Data‐driven modeling based on kernel extreme learning machine for sugarcane juice clarification

**DOI:** 10.1002/fsn3.985

**Published:** 2019-04-09

**Authors:** Yanmei Meng, Shuangshuang Yu, Hui Wang, Johnny Qin, Yanpeng Xie

**Affiliations:** ^1^ College of Mechanical Engineering Guangxi University Nanning China; ^2^ Energy, Commonwealth Scientific and Industrial Research Organisation Pullenvale Queensland Australia

**Keywords:** color value, extreme learning machine, gravity purity, particle swarm optimization, sugarcane juice clarification

## Abstract

Clarification of sugarcane juice is an important operation in the production process of sugar industry. The gravity purity and the color value of juice are the two most important evaluation indexes in the cane sugar production using the sulphitation clarification method. However, in the actual operation, the measurement of these two indexes is usually obtained by offline experimental titration, which makes it impossible to timely adjust the system indicators. A data‐driven modeling based on kernel extreme learning machine is proposed to predict the gravity purity of juice and the color value of clear juice. The model parameters are optimized by particle swarm optimization. Experiments are conducted to verify the effectiveness and superiority of the modeling method. Compared with BP neural network, radial basis neural network, and support vector machine, the model has a good performance, which proves the reliability of the model.

## 1 INTRODUCTION

Sugarcane juice clarification involves complex physical and chemical reactions. It is desirable to establish a mathematical model to analyze and control the clarification process, so that optimal final product be achieved. In control of complex industrial processes, there are generally two types of mathematical modeling approaches: modeling based on mechanism and modeling based on data‐driven. Abderafi and Bounahmidi (1999) used the adapted Peng–Robinson equation of state to estimate the boiling temperatures of industrial beet and sugar cane juices over a wide range of dry substance content. Jourani and Bounahmidi (2002) explained the reaction process of calcium phosphate in the first stage by combining the growth rate equation of crystal with the dissolution equation through the kinetic method. Mirsaeedghazi et al. (2010) proposed a mathematical modeling of mass transfer in the concentration polarization layer of flat‐sheet membranes during clarification of pomegranate juice. Cheng et al. (2011) used the data regression method to establish the quantitative relationship between calcium salt cations and acidic anions and constructed a mathematical model of calcium salt content and PH value. Hamerski, Silva, Corazza, Ndiaye, and Aquino (2012) presented a study of sugarcane juice carbonation and the evaluation of variable effects such as pH, carbonation time, and temperature on industrially relevant parameters for the quality of sugarcane juice. Three different batches of sugarcane juice were evaluated using a complete two‐level factorial design with central point performed in triplicate.

There are few studies on the mechanism model of the sugarcane juice clarification process. This is because the cane juice clarification process is a large time delay, multivariable coupling, and nonlinear process. It is extremely difficult to establish a complete mechanism model describing the clarification process which involves various complex physical and chemical reactions. Therefore, more and more researches turn to the process based on data‐driven approach, without the need for the mechanism description of the clarification process. By relying on the online and offline data of the monitoring system, a model predicting the development of the clarification process can be obtained through mathematical processing. Lin and Yang (2009) established an Elman network model which improved the dual heuristic dynamic programming to predict the neutralized pH value and the purified pH value of sugarcane juice. Song, Wu, Lin, and Liu (2012) used a generalized dynamic fuzzy neural network to predict the color value and alkalinity during the carbonation clarification process of sugarcane juice and obtained a satisfactory result. Sartori et al. (2017) proposed artificial neural network (Lambda NN) models to predict the effects of different variables on sugarcane juice color removal and sucrose content.

The above‐mentioned models of the clarification process focused on pH prediction, while more critical process parameters such as the gravity purity have not been involved. Moreover, these models are mainly based on the gradient descent method to update the model parameters (Al‐Batah, Mat Isa, Zamli, & Azizli, 2010). While the generalization performance of these models is good, there are some problems, such as slow training speed and easy to fall into the local optimum, which limits the application and development of the model (Kaya & Uyar, 2013; Mohammed, Minhas, Jonathan Wu, & Sid‐Ahmed, 2011).

In order to solve the problems of traditional learning machine of slow training speed and easy to fall into local optima, Huang, Zhu, and Siew (2006) proposed a new learning method, namely extreme learning machine (ELM). The ELM learning method has the advantages of less training parameters, very fast speed, and good generalization performance. Many researchers have applied ELM with different variants to solve different industrial problems. Wong, Wong, Vong, and Cheung (2015) used kernel‐based ELM and cuckoo search to model and optimize the performance of biodiesel engine. Farias et al. (2014) used extreme learning machine and bat algorithms to monitor product quality and provide fast and reliable product quality assessment of key process variables in second‐generation ethanol production. Mohammadi et al. (2015) proposed an extreme learning machine (ELM)‐based model for prediction of daily dew point temperature, and the model enjoys much greater prediction capability than SVM and ANN. Sadgrove, Falzon, Miron, and Lamb (2017) presented a color feature extreme learning machine (CF‐ELM) for fast object detection in pastoral landscapes, which takes three color inputs instead of the standard grayscale input.

Among the different applications of ELM, the kernel‐based ELM proved to have similar generalization performance to SVM while maintaining a much faster learning speed (Uçar & Özalp, 2017). Therefore, the kernel‐based ELM is employed in our research to tackle the large time delay and strong coupling problem in the sugarcane juice clarification process where establishing of mechanism model is difficult.

In our data‐driven model based on kernel extreme learning machine, four easy‐to‐measure variables in the sugarcane juice clarification process are selected as input, including the flow rate of the mixed juice, the intensity of sulfitation, the neutralization PH value, and the preliming PH value. Two difficult‐to‐measure variables are chosen as output, including the gravity purity of juice and the color value of clear juice. The parameters of the model are optimized by particle swarm optimization, and the effectiveness of the model is verified by experiment. To further evaluate the model performance on accuracy and time consuming, the results predicted from this model are also compared with those from other models such as BP, RBF and SVM.

## 2 KERNEL EXTREME LEARNING MACHINE AND DATA‐DRIVEN MODELING

### 2.1 Kernel extreme learning machine

The support vector machine will not fall into the local minimum point during the learning process which makes it more generalized than BP in training feedforward neural networks, and the fitting degree of the test data set is more reliable (Huang, 2014, Wang, Zheng, Yoon, & Ko, 2018). However, the support vector machine has limited applicability to the field of system modeling. For a complex system, it may be necessary to build multiple parallel networks, which will cause a long modeling period. In order to overcome the shortcomings of neural networks and support vector machine, inspired by biological learning, Huang et al. (2006) proposed a new learning method, namely extreme learning machine (ELM). Unlike the traditional neural network methods which usually are time consumed and easy to get overfitting results, the ELM does not need to tune parameters for its hidden layers, resulting in a faster training speed and an improved generalization performance (Huang, 2014; Lu, Du, Liu, Xia, & Yeap, 2017).

**Figure 1:**
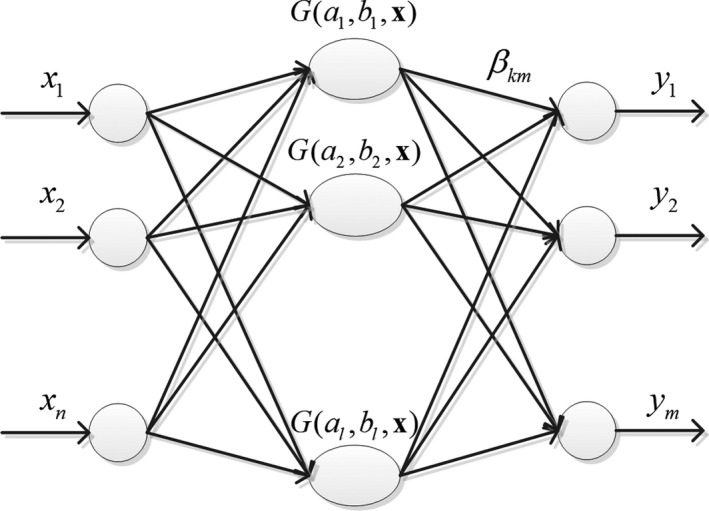
Network structure of extreme learning machine

The network structure of the extreme learning machine is shown in Figure 1.

Suppose a data sample set $S={\left\{\left(x_{i},y_{i}\right)\right\}}_{i=1}^{N}$has *N* samples, where $x_{i}={\left[x_{i1},x_{i2},\ldots ,x_{\mathrm{in}}\right]}^{T}\in R^{n}$is the input matrix, indicating that there are n input variables, and $y_{i}={\left[y_{i1},y_{i2},\ldots ,y_{\mathrm{im}}\right]}^{T}\in R^{m}$is the output matrix, indicating that there are m output variables. According to the structure principle of feedforward neural network, the mathematical model of input and output with the number of l hidden layer neurons can be expressed by Equation (1).

(1) $$f_{N}\left(x_{i}\right)=\sum_{k=1}^{l}\beta G(x)=\sum_{k=1}^{l}\beta G(〈a_{k},x_{i}〉+b_{k})$$

In the above formula, β is the network output weight; $a_{k}$ is the network input weight; $〈a_{k},x_{i}〉$is the inner product of $a_{k}$ and $x_{i}$; and $b_{k}$is the threshold of the kth hidden layer neuron. The activation function G(∙)of the hidden layer neurons can be any infinitely differentiable function such as sigmoid function, sine function, cosine function or compound function.

If the expression of Equation (1) is written as a matrix form consisting of *N* equations, it can be expressed as Equation (2).

(2) Hβ=Y

where H is the hidden layer output matrix.

(3) $$\begin{array}{c} H=H\left(a_{1},a_{2},\cdot \cdot \cdot ,a_{l},b_{1},b_{2},\cdot \cdot \cdot ,b_{l},x_{1},x_{2},\cdot \cdot \cdot ,x_{N}\right)\\ \begin{array}{cc} & = \end{array}{\left[\begin{array}{cccc} G\left(a_{1}\cdot x_{1}+b_{1}\right) & G\left(a_{2}\cdot x_{1}+b_{2}\right) & \cdots & G\left(a_{l}\cdot x_{1}+b_{l}\right)\\ G\left(a_{1}\cdot x_{2}+b_{1}\right) & G\left(a_{2}\cdot x_{2}+b_{1}\right) & \cdots & G\left(a_{l}\cdot x_{2}+b_{l}\right)\\ \vdots & \vdots & \cdots & \vdots \\ G\left(a_{1}\cdot x_{N}+b_{1}\right) & G\left(a_{1}\cdot x_{N}+b_{1}\right) & \cdots & G\left(a_{l}\cdot x_{N}+b_{l}\right) \end{array}\right]}_{N\times l} \end{array}$$

(4) $$\beta ={\left[\begin{array}{c} \beta_{1}^{T}\\ \vdots \\ \beta_{l}^{T} \end{array}\right]}_{l\times m},Y={\left[\begin{array}{c} y_{1}^{T}\\ \vdots \\ y_{N}^{T} \end{array}\right]}_{N\times m}$$

Unlike the traditional feedforward neural network which needs to adjust all network parameters in the training process to get the optimality, Huang et al. demonstrated that ELM input weights and hidden layer neuron thresholds can be randomly initialized prior to training and remain unchanged during training. The weight vector βconnected between the hidden layer and the output layer can be solved by Equation (5):

(5) $$∥H\left(a_{1},...,a_{l},b_{1},...,b_{l}\right)\overset{\wedge }{\beta }-Y∥=\min_{\beta }∥H(a_{1},...,a_{l},b_{1},...,b_{l})\beta -Y∥$$

The solution of the above formula is.

(6) $$\overset{\wedge }{\beta }=H^{+}Y$$

where $H^{+}$is the Moore–Penrose generalized inverse of the hidden layer output matrix H. It can be solved by various methods such as orthogonal projection, orthogonalization method, iterative method, and singular value decomposition. When using Moore–Penrose generalized inverse to solve Hβ=Y, it is a least squares solution and is easily overfitting in the case of large samples. By introducing the concept of kernel function into the extreme learning machine (Huang, Zhou, Ding, & Zhang, 2012), the kernel extreme learning machine (KELM) is formed. This can effectively avoid the original randomness of ELM, achieving faster training, and better generalization performance (Huang, 2014; Jian et al., 2017)

Replace the output matrix of the ELM with the corresponding kernel function, as shown in Equation (7):

(7) $${\mathrm{HH}}^{T}\left(i,j\right)=K\left(x_{i},x_{j}\right)$$

This leads to

(8) $${\mathrm{HH}}^{T}=\Omega_{\mathrm{ELM}}\;=\left[\begin{array}{ccc} K(x_{1},x_{1}) & \cdots & K(x_{1},x_{j})\\ \vdots & \ddots & \vdots \\ K(x_{N},x_{1}) & \cdots & K(x_{N},x_{N}) \end{array}\right]$$

(9) $$h\left(x\right)H^{T}=\left[\begin{array}{c} \;\;\;\;K(x,x_{1})\\ \;\;\;\;\;\;\;\;\;\;\;\;\;\;\;\;\;\;\;\vdots \\ \;K(x,x_{N})\; \end{array}\right]$$

Therefore, the output of KELM can be written as

(10) $$f_{N}\left(X\right)={\left[\begin{array}{c} K(x,x_{1})\\ \vdots \\ K(x,x_{n}) \end{array}\right]}^{T}{\left(\frac{I}{C}+\Omega_{\mathrm{ELM}}\right)}^{-1}Y$$

where I is the identity matrix, C is the penalty factor, and the Gaussian kernel function is chosen as the kernel function of the model.

### 2.2 Data‐driven modeling based on kernel extreme learning machine

#### 2.2.1 Input and output variables

There are many parameters that may have influence on the clarification of sugarcane juice. Table 1 lists the eight potential parameters that may have significant influence on the clarification process. To eliminate those parameters that have insignificant influence on the process so that to reduce the dimensionality of the data set required to be treated during the modeling, the principal component analysis (PCA) method is used. At the end, the parameters that have significant influence on the clarification process of the cane juice are extracted.

**Table 1:** Symbolic meaning table of parameter variable set for sugarcane juice clarification process

No.	Description
$n_{1}$	Mixed juice flow (t/hr)
$n_{2}$	Preliming valve opening (%)
$n_{3}$	Preliming PH value
$n_{4}$	Neutralization PH value
$n_{5}$	Clear juice PH value
$n_{6}$	Intensity of sulfitation (ml)
$n_{7}$	Floating color value
$n_{8}$	Clear juice brix (Bx)

By calculating the size of the eigenvalues of each variable and the cumulative contribution rate to the clarification, it is able to determine the features to be extracted by the PCA feature dimension reduction. Table 2 shows the contribution rate statistics of each variable calculated from a data set of 277 data samples, which were obtained by the experimental platform.

**Table 2:** Principal component contribution rate of parameters to sugarcane juice clarification process

No.	Contribution rate to the clarification process/%	Cumulative contribution rate/%
$n_{1}$	27.597	27.580
$n_{6}$	24.709	52.307
$n_{4}$	17.514	69.821
$n_{3}$	16.373	86.194
$n_{5}$	3.980	90.173
$n_{2}$	3.518	93.692
$n_{7}$	3.261	96.953
$n_{8}$	3.047	100

It can be seen from Table 2 that the cumulative contribution rate of the first four variables, $n_{1}$, $n_{6}$, $n_{4}$, and $n_{3}$, has already reached 86.19%, exceeding the usual requirement of 85% when using the cumulative variance contribution rate method (Niu, 2011). Therefore, the mixed juice flow (*x*_1_), the intensity of sulfitation (*x*_2_), the neutralization PH value (*x*_3_), and the preliming PH value (*x*_4_) are selected as input. Meanwhile, two unmeasurable parameters, the Gravity purity of juice (*y*_1_) and the Color value of clear juice (*y*_2_), are taken as output, as shown in Table 3.

**Table 3:** Input and output variable table

No.	Variables	Unit
$x_{1}$	Mixed juice flow	t/hr
$x_{2}$	Intensity of sulfitation	ml
$x_{3}$	Neutralization PH value	Null
$x_{4}$	Preliming PH value	Null
$y_{1}$	Gravity purity of juice	GP
$y_{2}$	Color value of clear juice	°St

#### 2.2.2 Parameter optimization

Particle swarm optimization (PSO) is a group intelligence global search optimization algorithm proposed by Kennedy in 1995 (Kennedy & Eberhart, 1995), which was inspired by the behavior of bird foraging groups. The particle swarm optimization algorithm has the characteristics of fast convergence, easy experimentation, and easy combination with other algorithms. It has been widely used in many fields, such as economic dispatch, robot application, signal processing, and image segmentation (Mahor, Prasad, & Rangnekar, 2009; Sengupta & Das, 2017; Suresh & Lal, 2017; Zhang, Gong, & Zhang, 2013). Therefore, the PSO is employed to optimize the parameters during the clarification process of the sugarcane juice.

The fitness function is used to evaluate the pros and cons of the particles. It has a direct impact on the algorithm optimization results. During the optimization process, each particle in the group moves according to its own fitness function value, rather than a random flight. In each iteration, the optimal value of the individual particles is updated by comparing its historical optimal value with the current state optimal value. $p_{i}=\left(p_{i1},p_{i2},\ldots ,p_{\mathrm{iD}}\right)$is used to denote the optimal position that individual particles can currently find, called "local optimum." After traversing each individual particle, the population global optimal value is updated by comparing the group history optimal value with the current state group optimal value. $p_{g}=\left(p_{g1},p_{g2},\ldots ,p_{\mathrm{gD}}\right)$ is used to indicate the optimal location that the group can currently find, called "global optimality."

The velocity and position iteration equations of the particle swarm algorithm are as follows:

(11) $$v_{\mathrm{id}}^{k+1}=\omega \cdot v_{\mathrm{id}}^{k}+c_{1}\cdot r_{1}\cdot \left(p_{\mathrm{id}}^{k}-x_{\mathrm{id}}^{k}\right)+c_{2}\cdot r_{2}\cdot \left(p_{\mathrm{gd}}^{k}-x_{\mathrm{id}}^{k}\right)$$

(12) $$x_{\mathrm{id}}^{k+1}=x_{\mathrm{id}}^{k}+v_{\mathrm{id}}^{k+1}$$

where *k* is the number of algorithm iterations, d=1,2,⋯,Dis the search solution in the D‐dimensional space, $x_{i}=\left(x_{i1},x_{i2},\cdots ,x_{\mathrm{id}}\right)$is the position vector of the particle *i* in the D‐dimensional search space, $v_{i}=\left(v_{i1},v_{i2},\cdots ,v_{\mathrm{id}}\right)$is the moving speed of the particle *i* position change, $p_{\mathrm{id}}^{k}$ is the optimal position of the individual particle after *k* iterations, $p_{\mathrm{gd}}^{k}$ is the optimal position of the group after *k* iterations. ω is the inertia factor, and $c_{1},c_{2}$ are the acceleration factors, and $r_{1},r_{2}$ are two random numbers between (0, 1).

The performance of the KELM‐based cane juice clarification process model will be affected by the penalty factor *C* and the kernel parameter σ. In this model, the prediction result RMSE of the sample data of the sugarcane juice clarification process is taken as the fitness function, and the optimal penalty factor *C* and kernel parameter σ can be obtained.

#### 2.2.3 Data‐driven model

**Figure 2:**
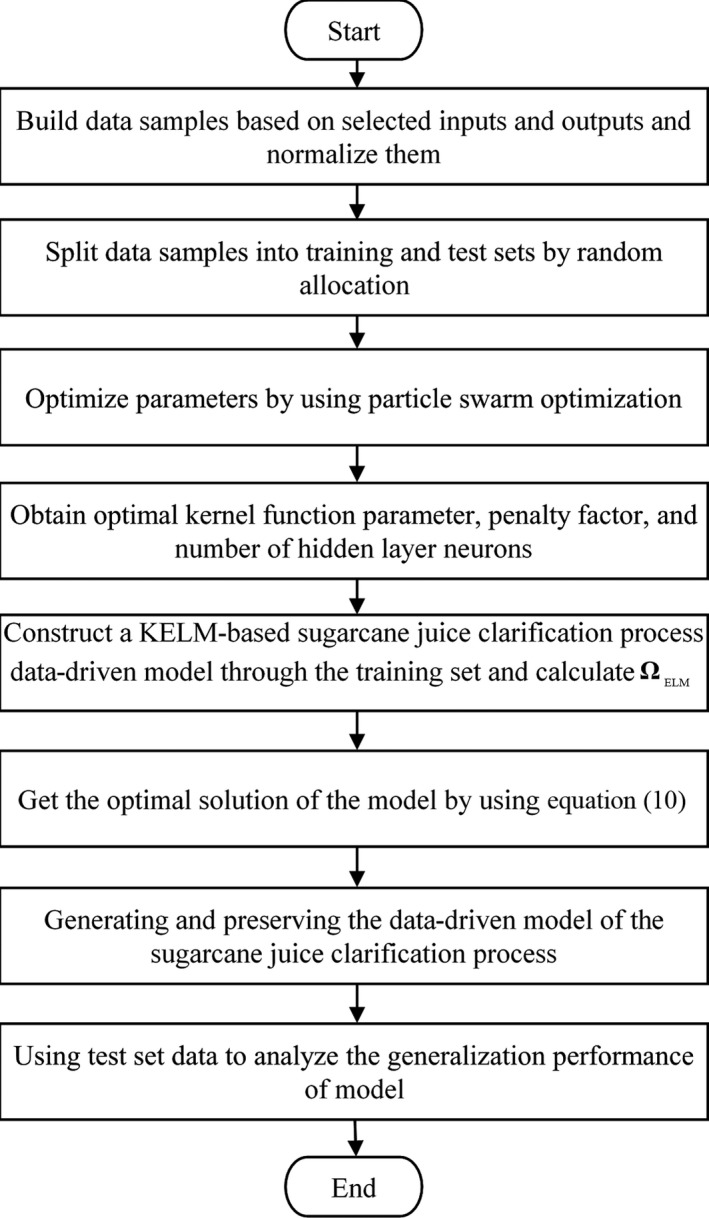
Flow chart of constructing the data‐driven model for sugarcane juice clarification process

The specific steps of construction of the data‐driven model based on KELM are shown in Figure 2. As mentioned previously, four parameters are selected as input, and two parameters are set as output. The Gaussian kernel function is chosen as the kernel function of the model.

## 3 EXPERIMENTAL RESULTS AND ANALYSIS

### 3.1 Model performance index

In order to evaluate the performance of the data‐driven model for sugarcane juice clarification, it is necessarily to define the model evaluation criteria. Suppose the actual value of the *i*‐th test sample with *m* test samples is expressed as $y_{i}$, the predicted value of the corresponding *i*‐th test sample is $\overset{\wedge }{y_{i}}$, and the mean value of the test sample is $\bar{y}=\frac{\sum_{i=1}^{m}y_{i}}{m}$. The performance of the data‐driven model for the sugarcane juice clarification process was evaluated by using the indexes shown in Table 4 (Malik, 2005).

**Table 4:** The model performance indexes and their definitions

Indexes of model performance evaluation	Formula
Root mean square error: RMSE	$\text{RMSE}=\sqrt{\frac{\sum_{i=1}^{m}{\left(y_{i}-{\hat{y}}_{i}\right)}^{2}}{m}}$
Mean absolute error: MAE	$\text{MAE}=\frac{\sum_{i=1}^{m}\left|y_{i}-{\hat{y}}_{i}\right|}{m}$
Determination coefficient: $R^{2}$	$R^{2}=\frac{\sum_{i=1}^{m}{\left({\hat{y}}_{i}-{\overset{¯}{y}}_{i}\right)}^{2}}{\sum_{i=1}^{m}{\left(y_{i}-{\overset{¯}{y}}_{i}\right)}^{2}}$

### 3.2 Experimental results and analysis

#### 3.2.1 Experimental platform

**Figure 3:**
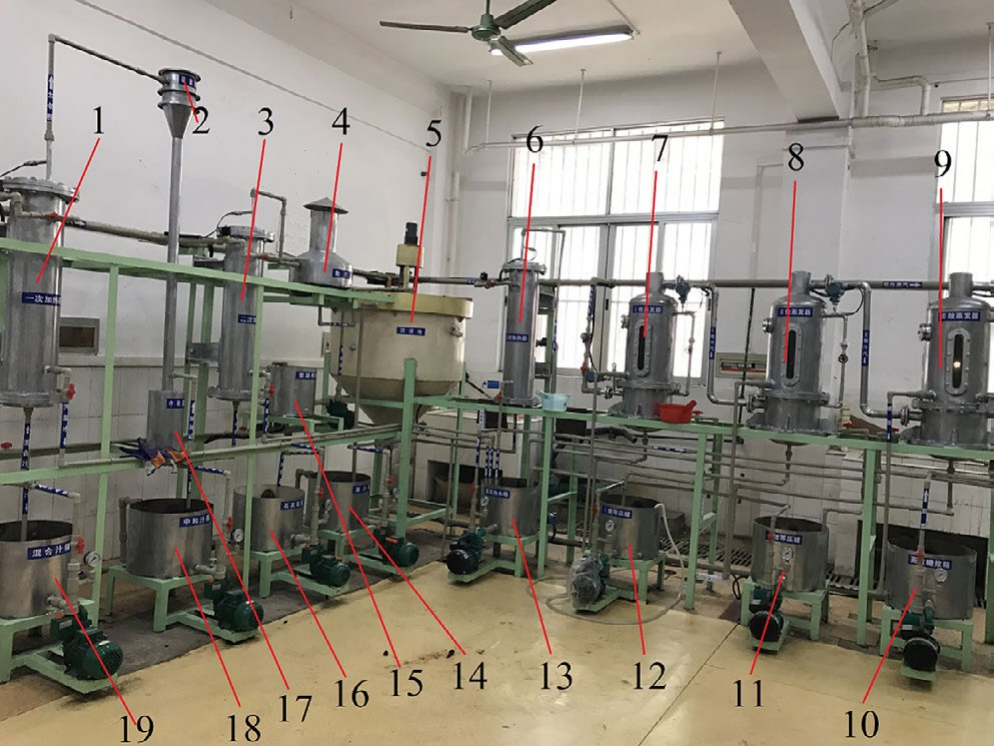
Sugarcane juice clarification experimental platform (1, 3, 6—heaters; 2—sulfitator; 4—diffusion box; 5—sedimentation tank; 7, 8, 9—evaporators; 10—syrup tank; 11,12—equal pressure tanks; 13—evaporating hot water tank; 14—clear juice tank; 15—flocculant tank; 16—lime milk tank; 17—neutralizer; 18—neutralizing juice tank; and 19—mixed juice tank)

In order to verify the data‐driven model of the sugarcane juice clarification process, a comprehensive experimental platform is developed. The platform mainly includes a sedimentation tank, some sugarcane juice tanks, an auxiliary part and a control valve part, as shown in Figure 3.

#### 3.2.2 Experimental result

The computer operating environment provided in this paper is Inter(R) Corei5, 2.4GHz, 4G memory, Windows 7 operating system, and MATLAB 2014a is used as the running computing software. During the clarification process of a batch of sugarcane juice using the experimental platform, 277 sets of data samples are obtained, and the data samples are normalized and divided into training set and testing set by random allocation. The number of the training set and testing set are 200 and 77, respectively. The training set is mainly used for training the optimization of model parameters by the group intelligence algorithm and the construction of the data‐driven model. The test set is used to test and analyze the data‐driven model. Four variables including the mixed juice flow, intensity of sulfitation, neutralization PH value, and preliming PH value are selected as the input, and two production indexes including the gravity purity of juice and the color value of clear juice are used as output. Some parameters of the data‐driven model, such as penalty factor *C* and kernel parameter σ, are optimized by PSO. The number of optimization iterations is set as 200, and the range of parameter optimization is set as [0, 1,024]. Using the root mean square error (RMSE) as fitness function, through a number of trial and error, the optimal number of hidden layer neurons is determined as 25. After the iteration is completed, the optimal parameter combination of the model is obtained. The optimization results are shown in Table 5.

**Table 5:** Optimal parameter results of data‐driven model for sugarcane juice clarification process

Optimization algorithm	Optimization time (s)	Root mean square error (RMSE)	Parameter optimization result	
			*C*	σ
PSO	38.4523	0.0562	112.76	71.38

After obtaining the optimal penalty factor and kernel function parameter, it is necessary to learn the training samples of the sugarcane juice clarification process and construct a data‐driven model. The RMSE, MAE, and *R* ^2^ are used to show the performance of the model. The training time represents the running time required by the CPU when the model is optimized using the training set to rebuild the model.

**Figure 4:**
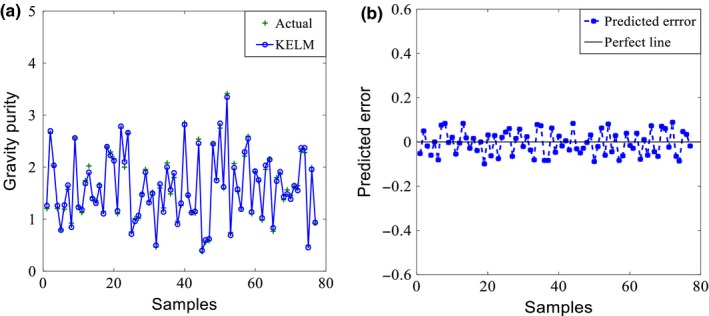
Test set prediction performance of data‐driven model with the gravity purity as output: (a) prediction result, (b) prediction error

Figure 4a shows the predicted gravity purity including the measured actual values for the purpose of comparison. Prediction fits the measurement very well. The prediction error fluctuation is small. The maximum error is 0.10 (see Figure 4b), accounting 6.21% of the average gravity of 1.61 of the data sample.

**Figure 5:**
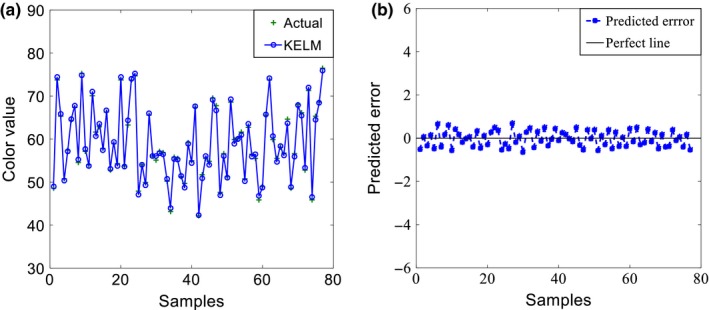
Test set prediction performance of data‐driven model with the color value as output: (a) prediction result, (b) prediction error

Figure 5 shows the prediction result and error when the color value is the model output. It can be seen that the maximum error is 0.69, accounting 1.17% of the average (59) of the color values.

Table 6 shows the model performance of the sugarcane juice clarification process. As can be seen in Table 6, the training time of the data‐driven model based on KELM is small (0.3325s), which indicates that the model has a high learning efficiency. The performance evaluation indicators, RMSE, and MAE are relatively low values. The coefficient of determination *R* ^2^ is 0.934 for predicting purity and 0.972 for predicting color value. Both are very close to 1, proving the excellent learning performance of the model.

**Table 6:** Data‐driven model performance of sugarcane juice clarification process based on KELM

Model output	Training time (s)	RMSE	MAE	$R^{2}$
Gravity purity of juice	0.3325	0.0764	0.0608	0.9337
Color value of clear juice		0.4593	0.2159	0.9722

#### 3.2.3 Comparison with other learning methods

**Figure 6:**
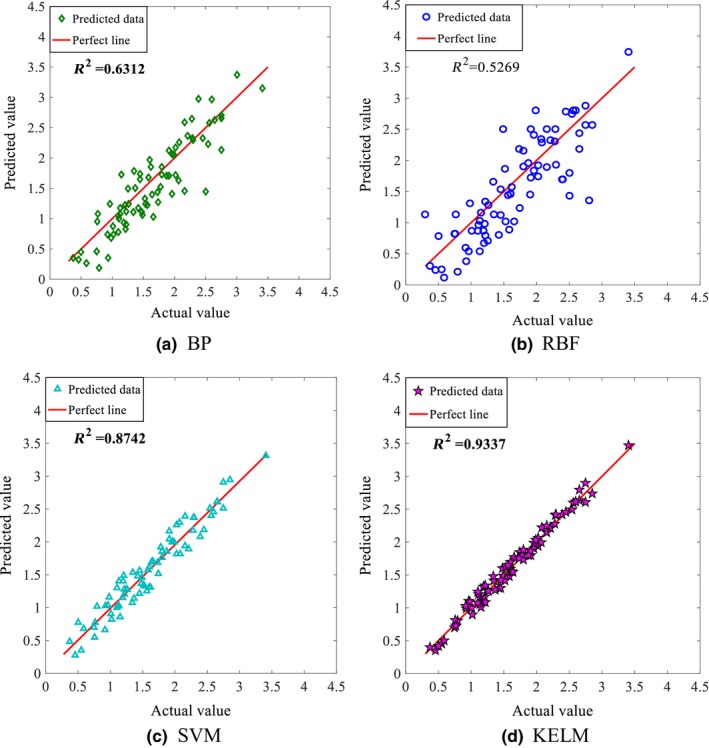
Comparison of coefficient of determination *R* ^2^ obtained from different data‐driven models with the gravity purity as output

**Figure 7:**
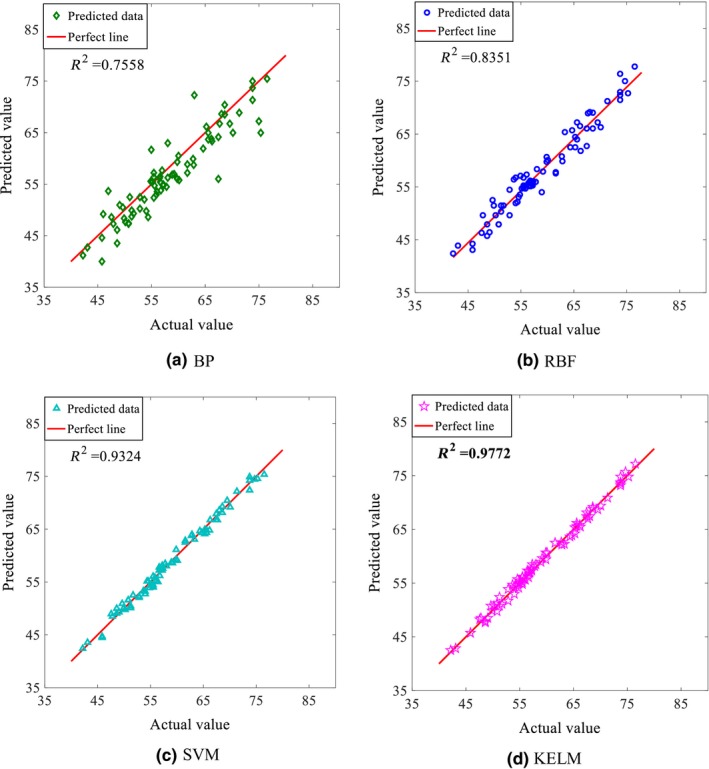
Comparison of coefficient of determination *R* ^2^ obtained from different data‐driven models with the color value as output

In order to verify the validity and superiority of the data‐driven model based on KELM for the cane juice clarification process, a number of models with different learning methods including BP, RBF, and SVM have been run using the same data set of 277 data samples that was used in the KELM‐based model to compare their performances. Figure 6 shows the performance comparison of the four models when the gravity purity of juice is chosen as output. Figure 7 shows the results of predicting the color value of clear juice. Tables 7 and 8 compare the performance indexes of different models in predicting gravity purity and color value, respectively.

**Table 7:** Model performance comparison of different models with the gravity purity as output

Algorithms	Training time (s)	RMSE	MAE	$R^{2}$
BP	3.0956	0.4465	0.2107	0.6312
RBF	4.3215	0.5327	0.2387	0.5269
SVM	0.5742	0.1812	0.1104	0.8742
KELM	0.3325	0.0764	0.0608	0.9337

**Table 8:** Model performance comparison of different models with the color value as output

Algorithms	Training time (s)	RMSE	MAE	$R^{2}$
BP	3.0956	1.1872	0.5399	0.7558
RBF	4.3215	1.0371	0.4832	0.8351
SVM	0.5137	0.6742	0.2691	0.9324
KELM	0.3325	0.4593	0.2159	0.9722

It is seen from Figures 6 and 7 that all models perform better in predicting the gravity purity than in predicting the color value. While the prediction accuracy and generalization performance of both BP and RBF are substandard with *R* ^2^ < 0.85, and large scattered data, the SVM and KELM models perform much better with *R* ^2^ of 0.93 and 0.97, respectively. Of the four models, the KELM performs the best with the highest *R* ^2^ value (0.97) and the most convergent data.

From Tables 7 and 8, it can be seen that while the training times of SVM and KELM are just 10% of that of BP and RBF for predicting purity and color value, the training time of KELM is the smallest among the four models. The error indexes, mean and absolute error (MAE), and root mean square error (RMSE) of the KELM are also the smallest among the four models, indicating the high predicting accuracy of the KELM model.

## 4 CONCLUSION

A data‐driven model based on kernel extreme learning machine has been established to predict the gravity purity of juice and the color value of clear juice which cannot be measured online during the large time‐delay nonlinear clarification of sugarcane juice. A comprehensive experiment platform has been built to verify the data‐driven model. Using the data obtained from the experiment, the principal component analysis method is used to extract four key variables that have significant influence on the clarification process as input. The two variables, gravity purity and color value, which are difficult to be measured are used as output. Optimizing of model parameters is achieved by particle swarm optimization. Finally, the model is validated by experimental data. The results show that the data‐driven model based on kernel extreme learning machine has good prediction performance and high reliability and efficiency. Comparative analysis of different models indicates the KELM model has superior performance.

## CONFLICT OF INTEREST

The authors declare no conflict of interest.

## ETHICAL STATEMENTS

This research does not involve any human or animal testing.
